# Unequal healthy ageing trajectories across Europe: socioeconomic development and age-related disease burden in the European Union and South-Eastern European countries

**DOI:** 10.1007/s11357-026-02273-0

**Published:** 2026-04-29

**Authors:** Nora Kovacs, Peter Piko, David Major, Vince Fazekas-Pongor, Zoltan Ungvari, Roza Adany

**Affiliations:** 1 Department of Public Health and Epidemiology, Faculty of Medicine, University of Debrecen, Debrecen, Hungary; 2 HUN-REN–UD Public Health Research Group, Department of Public Health and Epidemiology, Faculty of Medicine, University of Debrecen, Debrecen, Hungary; 3 Center for Epidemiology and Surveillance, National Laboratory for Health Security, Semmelweis University, Budapest, Hungary; 4 Institute of Preventive Medicine and Public Health, Semmelweis University, Budapest, Hungary; 5 Jozsef Fodor Center for Prevention and Healthy Aging, Semmelweis University, Budapest, Hungary; 6 Outpatient Clinic, Semmelweis University, Budapest, Hungary; 7 Doctoral College, Health Sciences Program/Institute of Preventive Medicine and Public Health, International Training Program in Geroscience, Semmelweis University, Budapest, Hungary; 8 Vascular Cognitive Impairment, Neurodegeneration and Healthy Brain Aging Program, Department of Neurosurgery, University of Oklahoma Health Sciences Center, Oklahoma City, OK USA

**Keywords:** Healthy ageing, WHO European Region, Socioeconomic development, Disease burden, Clusters

## Abstract

Although life expectancy has increased, many of the added years are lived in poor health, highlighting growing inequalities in ageing trajectories. Despite the increasing need for ageing-related health strategies, non-EU European countries remain underrepresented in comparative research, leaving limited evidence on healthy ageing disparities between EU and South-Eastern European (SEE) nations. This study explored variations in key health and socioeconomic indicators of societal ageing and age-related disease burden among individuals aged 70 and older across European countries, with a focus on EU and non-EU SEE nations. Data for 33 European countries were extracted from Global Burden of Disease 2021 study. Indicators included mortality, years lived with disability (YLD), healthy life expectancy (HALE), current health expenditure (HE), and Human Development Index (HDI). Hierarchical cluster analysis was used to classify EU countries, and SEE countries were analysed separately. Trends in mortality, YLD rates, and HALE were analysed using Joinpoint regression. Spearman’s rank correlation was used to assess relationships between health, socioeconomic factors, and disease burden. Western and Northern European countries showed the most favourable values for indicators, while SEE countries exhibited the highest all-cause and cardiovascular mortality and the lowest HALE and HDI values. Across all countries, strong negative correlations were observed between HE, HDI, HALE, and total mortality (r = -0.81, -0.82, and -0.92, p < 0.001; respectively). Regionally adjusted and coordinated policies are needed to reduce inequalities and promote healthy ageing across Europe. Such strategic integration is essential to narrowing the gaps in ageing trajectories and socioeconomic development between EU and SEE countries.

**Supplementary Information:**

The online version contains supplementary material available at 10.1007/s11357-026-02273-0.

## Introduction

Population ageing poses major challenges for health and social care systems, as well as for economies across Europe, particularly in countries with limited healthcare capacity and constrained resources. In response to Europe’s advanced demographic ageing, the concept of healthy ageing has become a key focus of health policy.

While the World Health Organization (WHO) emphasizes healthy ageing as the process of developing and maintaining functional ability that enables well-being in older age [1], the present study adopts a complementary, healthspan-oriented interpretation that is more directly aligned with population health assessment. In this context, healthy ageing is conceptualized as the extent to which gains in longevity are accompanied by delayed onset, reduced severity, and lower cumulative burden of age-related diseases and functional impairment. At the population level, this balance is reflected in indicators such as healthy life expectancy (HALE), years lived with disability (YLD), and cause-specific mortality, which jointly capture whether ageing trajectories are characterized by compression of morbidity or prolonged periods of ill health. Examining these indicators together allows assessment of how effectively different societies translate survival gains into preserved health and function, and how these patterns vary across regions with different levels of socioeconomic development and health system capacity [2].

In the period between 2000 to 2019, both life expectancy and HALE increased, but the increase in HALE was less than that of life expectancy, indicating that a greater proportion of added life years were spent in ill health [3–5]. By 2024, for the first time, people aged 65 years or older outnumbered those under 15 years in the WHO European Region [3]. Specifically, in the European Union (EU), 21.6% of the population was aged 65 years or older in 2024, and this proportion is projected to reach 30% by 2050. The old-age dependency ratio was approximately 34% in 2024, indicating that there were about three working-age (15–64 years per definition) individuals per person aged 65 and above. This ratio is projected to exceed 50% by 2050, further increasing the burden on health and social care systems [6]. This challenge is particularly acute in South-Eastern European (SEE) countries, where the demographic ageing is compounded by outward migration and declining fertility rates [7, 8].

Health profiles across European populations show regional heterogeneity, reflecting diverse demographic and ageing trajectories. Southern countries tend to have high life expectancy, but poorer overall health compared to Northern and Western counterparts. By contrast, Eastern European nations exhibit lower life expectancy, along with the highest mortality rates, especially in SEE region. Despite progress in several health and socioeconomic indicators over recent decades, this subregion continues to lag behind EU average and faces significant challenges in meeting Sustainable Development Goals [9, 10]. Previous research highlighted that reductions in infant mortality rates and increases in GDP per capita have been key drivers of longevity in EU accession candidate countries within the Balkan region [11]. Accordingly, addressing noncommunicable diseases (NCDs)—particularly cancer and cardiovascular disorders—remains essential to reduce age-related disease burden and promote healthy ageing in the SEE region [10].

In response to a decade of conflicts, the countries of South-Eastern Europe faced major challenges in rebuilding their political, economic, and social systems. To support stability and development in the region, the European Union and the international community launched the Stability Pact for South-Eastern Europe in 1999 as a conflict-prevention and reconstruction process [12]. It represented the first comprehensive and long-term strategy aimed at achieving stability in this conflict-affected region through closer integration into the European community [13]. A health component was added to the Pact in 2001, with the technical support of the WHO Regional Office for Europe. The South-eastern Europe Health Network (SEEHN) was thus established as a regional intergovernmental initiative to strengthen regional cooperation and coordination in public health and to improve health across the region. It currently brings together nine states of the region, including Albania, Bosnia and Herzegovina, Bulgaria, Montenegro, the Republic of Moldova, Romania, Serbia, North Macedonia and Israel.

Given the varying—although often overlapping—definitions of South-Eastern Europe, we adopt the SEEHN framework as a consistent basis for defining the region in this study. Although Israel has been a member of the SEEHN, it was not included in the present analysis. Israel joined the Network at a later stage (in 2011) and differs considerably from other SEE member states in terms of socioeconomic context and health system development, as well as its health indicator values are more aligned with those of Western European countries. Therefore, the present study focuses on the South-Eastern European countries that share more similar historical, social, and health system characteristics. In addition to Romania and Bulgaria, which are already EU member states, all these countries have been recognized as potential candidates for EU membership [13, 14].

Despite the growing need for comprehensive ageing-related health strategies, these countries—particularly the non-EU members—are often underrepresented in comparative ageing and health research, potentially undermining efforts to address the growing burden of age-related diseases and to support healthy ageing throughout Europe. This study aimed to explore variations in key health and socioeconomic indicators of ageing and age-related disease burden among individuals aged 70 and older in European countries, focusing on comparisons between EU and non-EU SEE nations. Our study aims to inform evidence-based regional health policy planning in support of healthy ageing.

## Methods

### Study design and data sources

This study utilized data from several publicly available databases. Age-related disease burden data for the population aged 70 years and above were retrieved from the Global Health Data Exchange (GHDx).

The Global Burden of Disease 2021 study provides comprehensive estimates for 371 diseases and injuries, as well as 88 risk factors by sex and age across 204 countries and territories [15]. GBD annual estimates from 1990 to 2021 are available through the publicly accessible GHDx platform (http://ghdx.healthdata.org/gbd-results-tool, accessed on 18 June 2025).

Data from 33 European countries were collected using the GBD 2021 database. We utilized death and years lived with disability (YLDs) rates associated with all causes and the major age-related chronic diseases including Alzheimer’s disease and other dementias, colon and rectum cancer, diabetes mellitus, ischaemic hearth disease, stroke, and tracheal, bronchus and lung cancer among the population aged 70 + years old. YLD refers to the years lived with any disability, weighted by the severity of the health condition [2, 16]. Mortality and YLD rates were calculated as age-specific rates per 100,000 population for individuals aged 70 years and above. All rates were expressed per 100,000 population.

In total, 33 countries including 27 EU member states and 6 non-EU SEE countries (Albania, Bosnia and Herzegovina, Moldova, North-Macedonia, Montenegro, and Serbia) were examined for this study.

The following health-related and socioeconomic indicators were selected for the analysis for 33 countries, covering the period 2011–2021 based on data availability.

### Health-related indicators

*Current healthcare expenditure per capita:* Current expenditures on health per capita expressed in international dollars at purchasing power parity (PPP). Data were obtained from the WHO Global Health Expenditure Database (GHED) (http://apps.who.int/nha/database).

*Healthy life expectancy at birth:* The average number of years a person is expected to live in full health, accounting for the years lived with illness or injury. Data were retrieved from WHO Global Health Observatory https://www.who.int/data/gho/data/indicators.

*Healthy life expectancy at age 60:* The average number of years a person (at age 60) can expect to live in full health, based on current rates of morbidity and mortality. Data were retrieved from WHO Global Health Observatory https://www.who.int/data/gho/data/indicators.

### Socioeconomic indicators

*Human Development Index (HDI):* It was used as an indicator of socioeconomic development of a country, which is a composite index measuring average achievement in three dimensions of human development: long and healthy life, knowledge and a decent standard of living. The HDI is calculated based on four components: life expectancy at birth, mean years of schooling for adults aged 25 years and older, expected years of school education, and gross national income (GNI) per capita [17]. HDI data were obtained for each country from the UNDP's Human Development Data Portal (United Nations Development Programme; http://hdr.undp.org/en/data, accessed on 16 June 2025). The HDI ranges from 0 to 1, with higher scores reflecting a higher degree of socioeconomic development.

*Urban population (% of total population):* It refers to proportion of people living in urban areas in the percentage of total population as defined by national statistical offices.

*Population ages 65 and above (% of total population):* It refers to the population ages 65 and above as a percentage of the total population.

Data on urban population and population aged 65 and above were obtained from Population Division of the Department of Economic and Social Affairs of the United Nations.

### Statistical analysis

Since the European Union is not a homogeneous region, EU member states were grouped using agglomerative hierarchical cluster analysis based on 2021 data. The clustering was performed using a subset of core indicators related to healthy ageing (HDI, current health expenditure in PPP (HE), and HALE at age 60), while additional indicators were used to compare the resulting groups with SEE countries. Prior to clustering, all variables were standardized to ensure comparability across different measurement scales. Hierarchical cluster analysis was conducted using the complete linkage method with squared Euclidean distance. This approach emphasizes maximum separation between clusters, allowing for clearer distinction between country groups based on health and socioeconomic indicators. The optimal number of clusters was determined to be three, and the resulting cluster structure was visualized using a dendrogram. As a sensitivity analysis, latent profile analysis (LPA) was performed using continuous standardized indicators to assess the robustness of the clustering structure.

The non-EU SEE countries comprised six countries including Albania, Bosnia and Herzegovina, Montenegro, Moldova, Serbia and North Macedonia. Additionally, a sensitivity analysis was performed that included Israel among the SEE countries to enable comparison of the indicators, with the results presented in the Supplementary File.

Descriptive statistics with means and standard deviations were computed for all health and socioeconomic indicators for each country group. To assess differences between country groups in health and socioeconomic parameters, non-parametric Kruskal–Wallis tests were conducted. For pairwise comparisons, Mann–Whitney U tests with Bonferroni correction were conducted to determine whether differences between specific country groups were statistically significant.

Trend analysis of mortality, YLD rates and HALE was conducted using the Joinpoint regression analysis during 2011–2021. All joinpoint analyses were conducted using Joinpoint Regression Program (Version 5.4.0 – April 16, 2025, Statistical Methodology and Applications Branch, Surveillance Research Program, National Cancer Institute) [18]. The average annual percent change (AAPC) was estimated with log-transformation.

To examine temporal patterns in aging trajectories in the EU and SEE countries, latent class trajectory analysis was performed using healthy life expectancy at age 60 across the study period. HALE values were standardized to ensure comparability. Model fit was evaluated based on Akaike (AIC) and Bayesian (BIC) information criteria, and log-likelihood values. Posterior probabilities were used to assess classification quality.

The correlation between health-related and socioeconomic factors and disease burden was examined using Spearman’s rank correlation test. The results were plotted in scatter plots. Statistical analyses were conducted using Stata IC version 17.0 (Stata Corp., College Station, TX, USA). The *lcmm* package in R was used for latent class trajectory model, while latent profile analysis was conducted using generalized structural equation modelling in Stata. A p-value of less than 0.05 was considered statistically significant.

## Results

**Fig. 1:**
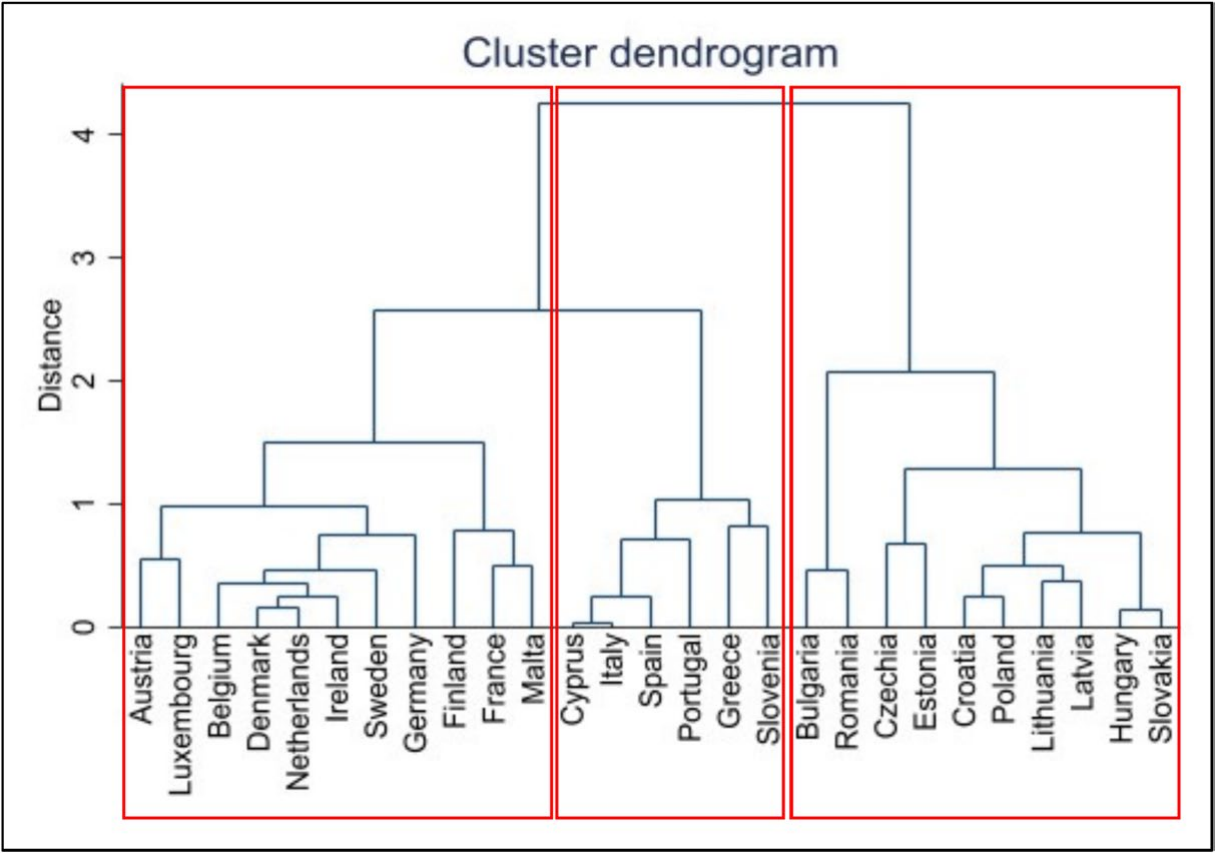
Hierarchical clustering of EU member states in terms of health expenditure, socioeconomic development and healthy life expectancy at 60 based on relevant data from 2021. The country clusters are separated with red lines

In this study, three clusters of EU countries were identified in the dendrogram. Cluster 1 included eleven countries exhibiting similar characteristics. These countries included Western and Northern European countries (Austria, Belgium, Denmark, Germany, Finland, France, Ireland, Luxembourg, Netherlands, Sweden) and Malta, all have high development based on the HDI, high health expenditure and high HALE at 60. Custer 2 consisted of six countries (Cyprus, Spain, Greece, Italy, Portugal, Slovenia) mostly Mediterranean countries. The third cluster included 10 countries mainly from Central and Eastern Europe (Bulgaria, Czechia, Croatia, Estonia, Poland, Hungary, Slovakia, Lithuania, Latvia, and Romania) with lower HDI, lowest health expenditure and low HALE at 60 (Fig. 1). Sensitivity analysis using LPA showed results largely consistent with the hierarchical clustering solution, supporting cluster stability. The only discrepancy was observed for Malta, which was assigned to the Cluster 2 in the LPA model, reflecting its intermediate profile (Supplementary Figure S1).

Table 1 presents the differences in health status and socioeconomic characteristics between EU and non-EU SEE countries (Albania, Bosnia and Herzegovina, Montenegro, Moldova, Serbia and North Macedonia). Cluster 1 consistently demonstrated the most favourable outcomes, with significantly higher current health expenditure, HDI and its components, as well as healthy life expectancy at birth and at age 60 compared to SEE countries. The indicators in Cluster 1 were generally more favourable than those in EU Clusters 2 and 3, although there were some exceptions—for example, in terms of mortality rates from ischemic heart disease and lung cancer. Cluster 1 and 2 showed significantly lower rates of all-cause mortality, and ischemic heart disease and stroke mortality than SEE countries, whereas they reported significantly higher mortality rates from Alzheimer’s disease. Differences in YLD rates were less marked. SEE countries exhibited a significantly higher diabetes burden than Cluster 1, while their colorectal cancer YLD rates were significantly lower than in all three EU clusters. The SEE region also showed a smaller share of older adults and lower levels of urbanization compared with the EU clusters, although only Cluster 1 differed significantly from SEE countries in this respect (Table 1).

**Table 1:** Means (SD) and pairwise comparisons of health and socioeconomic characteristics across EU clusters and SEE countries

Indicator	Cluster 1	Cluster 2	Cluster 3	SEE countries	p-value^#^
	**Mean (SD)**				
Current health expenditure per capita, in international dollar (PPP)	7097.86 (837.77)*	4253.02 (624.63)*	3115.41 (640.42)*	1817.33 (582.32)	< 0.001**
Human Development Index	0.94 (0.02)*	0.9 (0.02)*	0.87 (0.03)*	0.8 (0.02)	< 0.001**
Life expectancy at birth (years)	81.87 (0.53)*	81.46 (1.18)*	74.78 (2.05)	73.39 (2.57)	< 0.001**
Expected years of schooling	17.61 (1.75)*	17.44 (1.27)*	15.78 (0.79)*	14.38 (0.84)	0.001**
Mean years of schooling	12.66 (0.69)	11.39 (1.22)	12.72 (0.83)	11.23 (1.02)	0.014**
GNI per capita in international dollar (PPP)	67,313.76 (13,135.47)*	42,635.57 (6214.02)*	38,654.61 (5259.18)*	19,715.74 (3597.34)	< 0.001**
HALE at birth (years)	70.15 (0.65)*	69.97 (0.98)*	64.97 (1.47)	64.27 (1.84)	< 0.001**
HALE at 60 (years)	18.19 (0.47)*	18.02 (0.71)*	14.4 (0.86)	13.54 (1.07)	< 0.001**
Death rate per 100,000 people (aged 70 +)					
All-cause mortality	5154.03 (430.2)*	5689.67 (568.86)*	8077.55 (1314.1)	9340.13 (1590.13)	< 0.001**
Alzheimer's disease and other dementias	443.74 (66.76)*	462.98 (103.97)	324.99 (40.35)	260.81 (53.38)	< 0.001**
Colon and rectum cancer	172.66 (28.93)	178.02 (24.75)	214.93 (31.84)*	161.11 (44.43)	0.023**
Diabetes mellitus	108.05 (35.34)	164.06 (85.86)	138.53 (51.87)	217.38 (129.92)	0.298
Ischemic heart disease	756.11 (231.64)*	711.45 (191.73)*	1945.87 (425.02)	2137.5 (426.18)	< 0.001**
Stroke	402.45 (45.84)*	612.69 (174.7)*	1079 (571.95)	2030.53 (705.38)	< 0.001**
Tracheal, bronchus, and lung cancer	230.52 (54.48)	216.87 (54.43)	216.26 (55.88)	227.36 (84.36)	0.769
YLD rate per 100,000 people (aged 70 +)					
All-cause YLD	27,235.41 (1161.77)	27,534.63 (1107.56)	28,310.03 (829.1)	27,550.1 (841)	0.161
Alzheimer's disease and other dementias	1969.92 (378.65)	2128.79 (306.53)*	1839.99 (146.85)	1645.44 (197.69)	0.030**
Colon and rectum cancer	175.96 (55.81)*	156.4 (25.29)*	134.06 (32.49)*	84.07 (22.85)	0.001**
Diabetes mellitus	1421.49 (357.25)*	2035.25 (398.1)	2012.84 (466.25)	2578.85 (713.99)	0.003**
Ischemic heart disease	436.34 (93.38)*	372.86 (101.06)*	605.28 (81.24)	588.51 (46.06)	0.001**
Stroke	957.69 (340.49)	747.36 (222.32)	1191.76 (265.66)	1101.54 (228.37)	0.048**
Tracheal, bronchus, and lung cancer	53.06 (12.32)	46.65 (12.08)	44.86 (10.6)	43.22 (16.13)	0.488
Population ages 65 and above (% of total population)	19.4 (2.58)	20.65 (3.68)	20.1 (1.46)	17.69 (2.93)	0.302
Urban population (% of total population)	83.72 (12.51)*	70.26 (9.56)	65.47 (8.25)	56.44 (9.03)	0.001**
Countries (n = 33)	Austria	Cyprus	Bulgaria	Albania	
	Belgium	Spain	Czechia	Bosnia and Herzegovina	
	Germany	Greece	Estonia	Moldova	
	Denmark	Italy	Croatia	North Macedonia	
	Finland	Portugal	Hungary	Montenegro	
	France	Slovenia	Lithuania	Serbia	
	Ireland		Latvia		
	Luxembourg		Poland		
	Malta		Romania		
	Netherlands		Slovakia		
	Sweden				
	n = 11	n = 6	n = 10	n = 6	

^*^Statistically significant. Pairwise comparisons are based on Mann–Whitney U tests with Bonferroni correction (SEE countries vs. individual clusters). The adjusted significance threshold was p < 0.016 (0.05/3)

^#^p-values based on the Kruskal–Wallis test. **p-values (significant at p < 0.05)

HALE: healthy life expectancy, YLD: years lived with disability

**Fig. 2:**
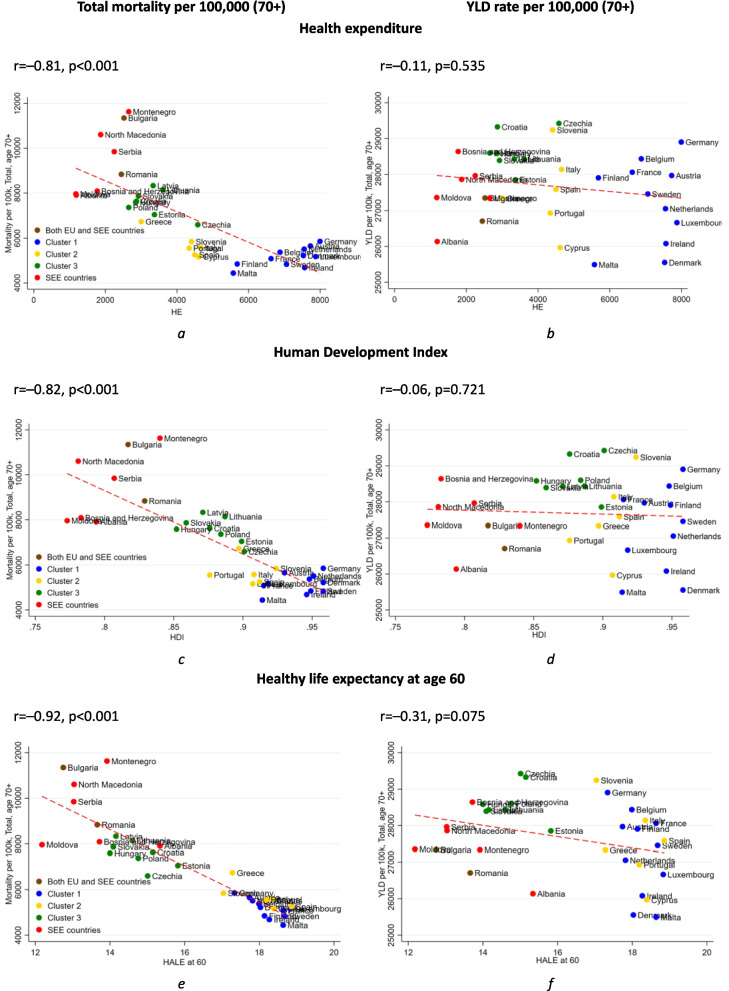
Spearman correlations between health and socioeconomic characteristics and mortality and YLD rates among aged 70 + for European countries (n = 33) in 2021. HE: Health expenditure, HDI: Human Development Index, HALE: Healthy life expectancy

Figure 2 (a, c, e) shows the Spearman correlation coefficients, which revealed strong and consistent relationships between HE, HDI and HALE at age 60 and mortality outcomes, with higher values of these indicators negatively correlated with total mortality among individuals aged 70 and above (r = −0.81, −0.82, and −0.92, respectively; p < 0.001). No significant correlations were found between health and socioeconomic indicators and YLD rates (Fig. 2 b, d, f). Inverse negative correlations were observed with ischemic heart disease and stroke mortality. By contrast, positive correlations were found for Alzheimer’s disease mortality with a correlation coefficient ranging from 0.68 to 0.75 (p < 0.001), indicating that countries with longer and healthier life expectancy tend to report higher dementia-related deaths, likely reflecting greater diagnostic capacity and survival into older ages (Supplementary Figure S2).

**Fig. 3:**
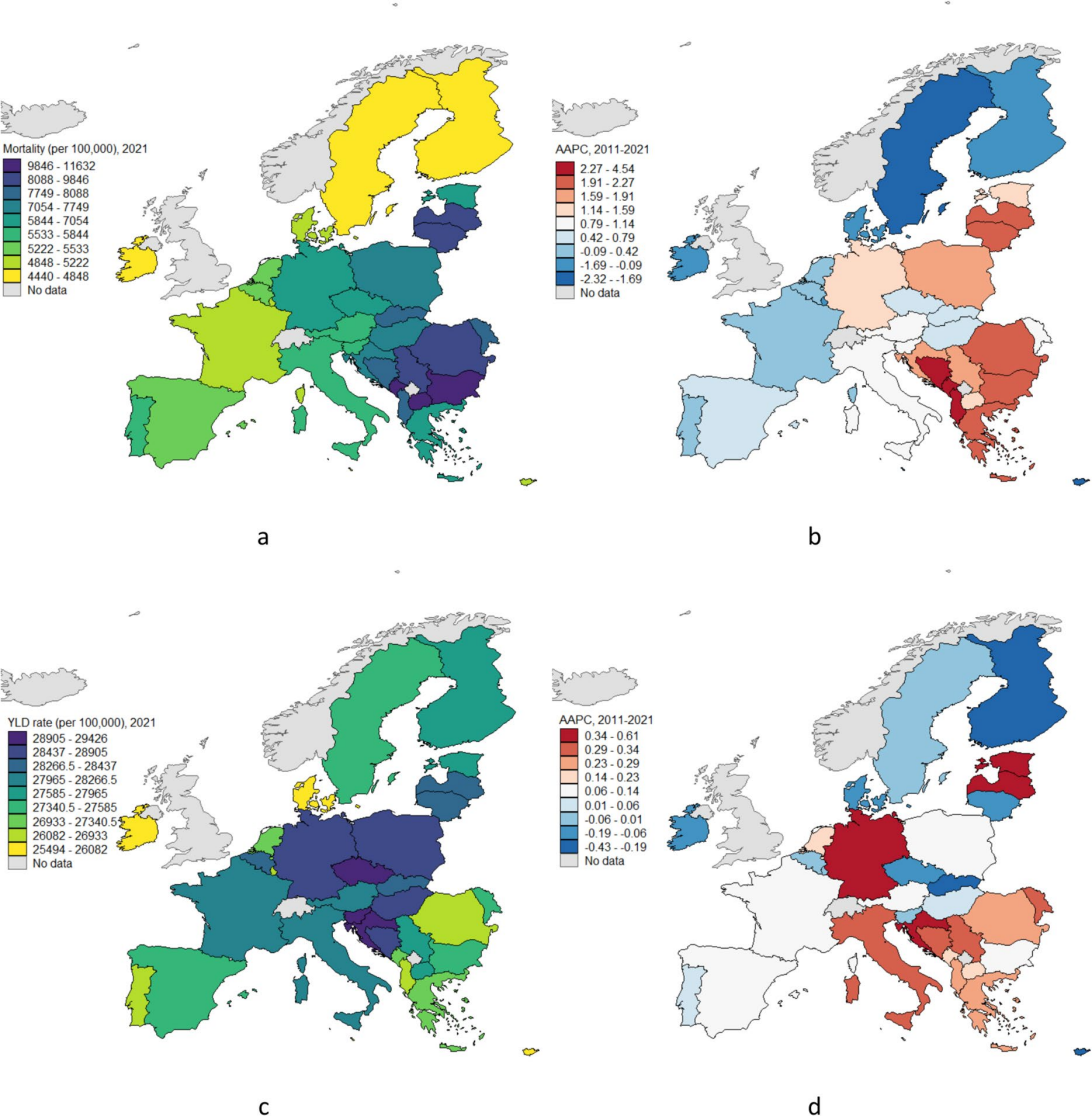

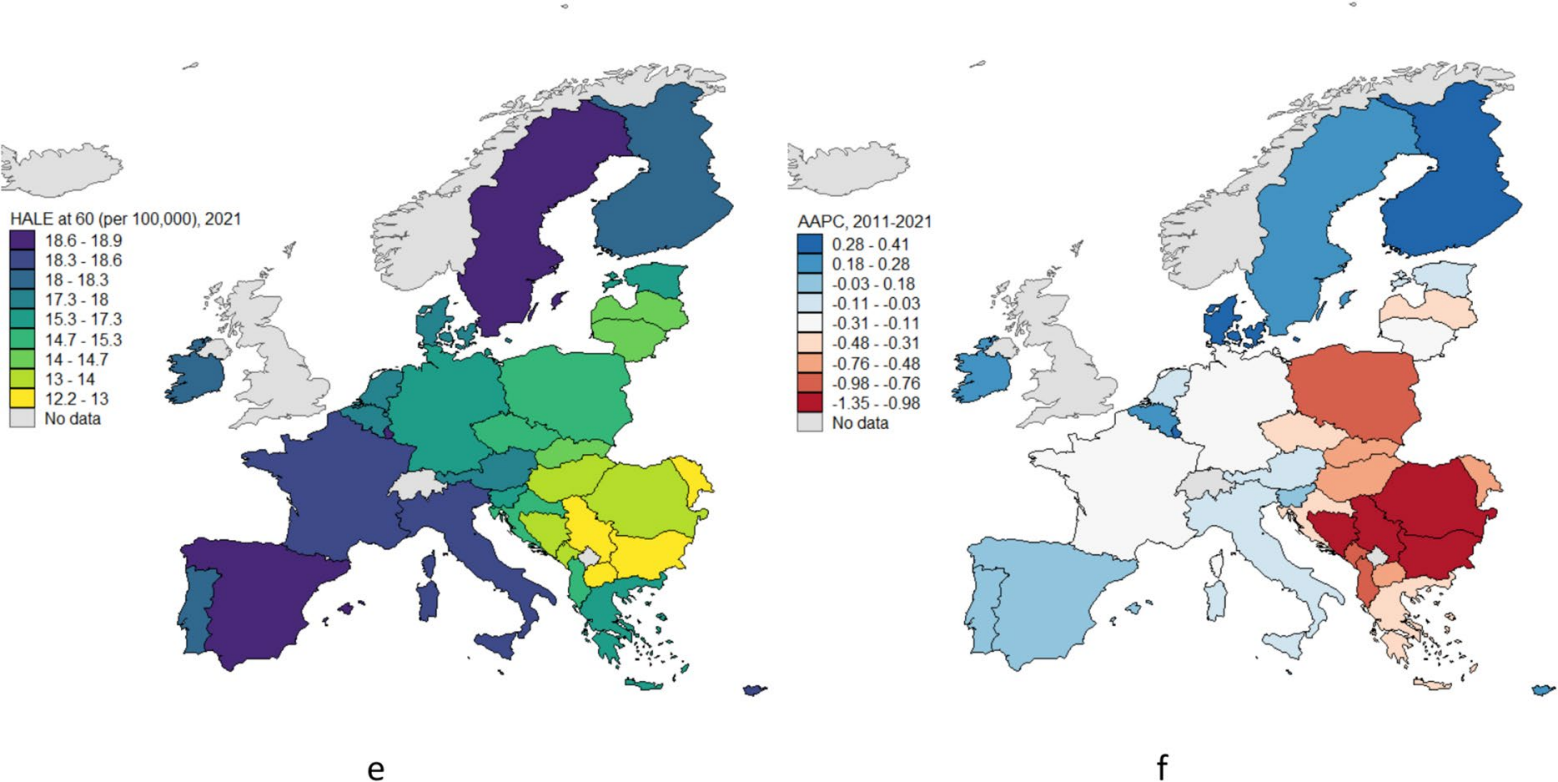
Mortality (**a**), YLD rates (**c**) per 100,000 among adults aged 70 +, and HALE at age 60 (**e**) in 2021, with average annual percentage change (**b**, **d**, **f**) from 2011 to 2021 across 33 European countries

Geographical disparities in mortality, disability, and healthy life expectancy at age 60 were observed among older adults across European countries (Fig. 3). In 2021, the highest mortality rates were found in SEE countries, particularly in Bulgaria (11,349 per 100,000), Montenegro (11,632 per 100,000) and North Macedonia (10,608 per 100,000), while the lowest rates were found in Northern and Western Europe. Between 2011 and 2021, the AAPC in mortality derived from joinpoint regression indicated an unfavourable upward trend in mortality in most SEE countries.. The YLD rate was higher in Central and Eastern Europe, with a modest increase over time. In contrast, HALE at age 60 remained substantially lower in CEE and SEE countries than in Western and Northern Europe.(Fig. 3). Although many countries exhibited increasing trends in HALE prior to 2019, more favourable or stable overall trends were generally observed in Northern and Western European countries, whereas most SEE and CEE countries experienced stagnation or significant declines over the study period, which may partly reflect the impact of the COVID-19 pandemic in the later years. Country-specific trends in mortality, YLD rates among adults aged 70 years and older and HALE at 60 estimated by Joinpoint regression are provided in Supplementary Table S2, S3 and S4.

The trajectory analysis of HALE at age 60 identified two classes based on AIC and BIC values (Supplementary Table S5-S6). Class 1 (n = 15) comprised primarily Central and Eastern Europe and Western Balkan countries and was characterized by consistently lower HALE over the study period (2011–2021). Class 2 (n = 18) included the remaining countries (Supplementary Figure S3). The trajectories demonstrated a clear and persistent separation between the two groups over time.

## Discussion

Effective interventions to support healthy ageing depend on health assessments for older adults, allowing personalized approaches to accurately address age-specific care needs in diverse demographic contexts [19, 20]. In this study, we aimed to identify distinct clusters of European countries based on socioeconomic and healthy ageing indicators, and to compare SEE nations in order to inform targeted, region-specific health policies. By integrating summary health measures such as life expectancy and HALE with socioeconomic and health system indicators derived from multiple data sources, our analysis provides a policy-relevant perspective that is directly interpretable within the framework of European health systems and regional cooperation initiatives such as SEEHN. Our analysis revealed that non-EU SEE countries had significantly lower HDI, and health expenditure per capita compared to EU countries. These disparities translated into notably poorer outcomes for life expectancy and HALE at birth and at age 60, underscoring that longevity is often accompanied by prolonged years of poor health rather than an increased healthy lifespan [21–23].

High mortality and YLD from NCDs were observed among older adults in both EU and non-EU countries. While the overall death rate from CVD has declined in the EU in recent decades, mainly attributed to improved primary prevention, risk factor management and care [3], marked east–west gaps persist [24–26]. Central and Eastern Europe and SEE countries continue to report substantially higher mortality due to ischemic heart disease and stroke. Stroke mortality was over four times higher in the SEE countries than in the best-performing EU countries. A recent study found that ischemic heart disease mortality also occur at younger ages, the age-standardized premature (≤ 65 years) IHD mortality in South-Eastern Europe remains about twice the Western European average, largely driven by a limited set of modifiable risk factors [27]. Comprehensive strategies should integrate individual and population-level approaches through multi-sectoral action addressing health systems and social determinants, as well as emphasizing the prevention of CV risk factors over a life course [25].

Similarly, the diabetes burden was significantly higher in SEE countries. Cross-national studies indicate that the burden of diabetes in Eastern and Central Europe is increasing more rapidly than in many Western European countries, with Bosnia and Herzegovina and North Macedonia among the countries with the highest type 2 diabetes burden in the WHO European Region [28]. This disparity is largely attributed to lower socioeconomic development, limited health-care resources, and slower adoption of advanced diabetes management technologies, factors that hinder effective prevention and treatment of diabetes [28, 29].

In contrast, Alzheimer’s disease and dementia showed lower mortality and YLDs in SEE countries, a pattern that likely reflects substantial underdiagnosis and underreporting of cognitive decline in lower-resource settings rather than a genuinely lower disease burden [30]. These disparities are consistent with regional gaps in diagnostic capacity, access to specialist care, and long-term dementia services [31, 32]. Indeed, a cross-sectional study across 19 European countries estimated that nearly 50% of individuals with probable dementia remain undiagnosed, with underdiagnosis being most pronounced in countries with weaker health systems [32]. At the same time, the positive association observed between socioeconomic development, HALE, and dementia-related mortality represents a well-described epidemiological paradox. In countries with higher life expectancy and stronger health systems, a greater proportion of individuals survive into advanced ages at which dementia becomes prevalent, and diagnostic ascertainment as well as cause-of-death attribution are more complete [33]. Conversely, lower dementia mortality in SEE countries likely reflects a combination of competing risks from premature cardiovascular mortality, limited access to cognitive assessment, and underdeveloped long-term care infrastructure [34]. Taken together, these findings underscore that dementia mortality statistics are strongly shaped by survival patterns and health system capacity and should therefore be interpreted as indicators of diagnostic and care infrastructure rather than standalone measures of population-level cognitive health.

Our results also highlighted that sociodemographic development may have a significant impact on health outcomes, beyond the influence of health spending alone, reflecting the strong gradient between development and longevity in Europe and globally [11, 35, 36]. This highlights the importance of comprehensive, cross-sectoral strategies for healthy ageing that combine poverty reduction, education, and risk-factor control with health-system investments [35, 36].

The relative stability of overall YLD rates across regions, despite pronounced differences in mortality and healthy life expectancy, suggests that reductions in fatal disease burden have not been matched by equivalent gains in disability prevention and functional preservation [5]. This pattern indicates that while primary prevention and acute care may reduce mortality, insufficient emphasis is placed on secondary prevention, rehabilitation, and long-term management of chronic conditions in older age. As a result, survival gains may translate into prolonged periods of living with disability, particularly in lower-resource settings [37]. Policies aimed at healthy ageing must therefore extend beyond mortality reduction to include systematic investment in rehabilitation services, chronic disease self-management, and integrated long-term care.

Interestingly, EU cluster 3 and SEE countries shared similar profiles for several indicators such as life expectancy, HALE, mortality due to major chronic diseases, and YLD rates, suggesting a close alignment between less affluent EU member states and non-EU SEE countries. Nonetheless, gaps remained in HDI, GNI per capita, and diabetes-related YLD rate, highlighting the need for targeted investment in primary care, NCD prevention, and diagnostic capacity for metabolic and cognitive disorders. These findings reassure the results of previous research showing that despite some progress towards EU health and health care policies, this subregion continues to face diverse public health challenges, including life expectancy, CVD and cancer mortality, and public-sector health spending [10]. Furthermore, a comparative study on population health dynamics between Western European and Balkan countries demonstrated that although the overall population health has improved considerably, Baltic and Balkan countries still lag behind on numerous key health indicators and several areas should be strengthened [7].

Although YLD rates have remained stable across regions, disease-specific YLDs especially for diabetes, stroke, and dementia, varied markedly between clusters, indicating that aggregated disability estimates may mask substantial inequalities between disease groups and countries. In line with prior work, gains in life expectancy without sufficient morbidity compression suggest that older adults in less affluent settings spend more years with disability [38]. Previous regional studies have confirmed that disability levels persist at older ages, primarily due to functional decline, injuries, and chronic conditions [2, 39]. Therefore, CVD risk-factor management, diabetes prevention and control, and secondary prevention for stroke remain critical to reducing lost healthy years—priorities emphasized by WHO Europe given the substantial contribution of NCD risk factors to death and disability [40]. Interventions addressing both lifestyle and socioeconomic determinants have a predictable impact for healthy ageing [39] and measurable mortality benefits in Europe [40].

The overlapping membership of Bulgaria and Romania underscores that, despite EU integration, these countries exhibit health and ageing-related outcomes closer to non‑EU SEE countries than to the more affluent EU average. Both nations have among the lowest life expectancies, high premature mortality from cardiovascular diseases, and relatively low per-capita health expenditure among EU member states [41].

Dasic et al. reported a steady, gradual rise in HDI across the Western Balkans, indicating continued progress in human development, and consequently, in population health. They also note, that HDI rankings do not always match the values of its components, suggesting that poverty reduction, sustained economic growth, and improved education and health care are all crucial for better human development [35]. These findings support our observation that HDI disparities between SEE and EU clusters contribute to differences in healthy life expectancy and highlight the need for policies that reduce social and educational inequalities alongside investments in health care to gain improvements in healthy ageing.

Based on data from 2011 to 2021, the findings indicate that the older population in South-Eastern European countries was disproportionately affected by the COVID-19 pandemic. Elevated mortality rates and the stagnation or decline in healthy life expectancy between 2011 and 2021 suggest that the pandemic intensified existing health and social inequalities across the region [42, 43].

Our results confirm that European countries exhibit considerable variation in their ageing trajectories [44]. The WHO Global Strategy on Ageing and Health [45] and the Decade of Healthy Ageing [46] provide comprehensive frameworks for action, but their implementation is hindered by varied cultural contexts, national capacities, and the limited availability of comparable data. To overcome these barriers, tools such as country clustering based on development and health indicators can support meaningful comparison and inform targeted, evidence-based policy responses that are aligned with national capacities [44]. Several regional policy frameworks have been developed for the SEE region. The SEE 2030 Strategy [47] places particular emphasis on accelerating convergence with the European Union, while promoting inclusive and sustainable development through reducing inequalities. The Strategy also supports regional cooperation in achieving the UN Sustainable Development Goals and the 2030 Agenda. In addition, a comprehensive policy analysis by the OECD [48] provides an evidence-based assessment of key policy areas in the Western Balkan (WB6) economies (Albania, Bosnia and Herzegovina, Kosovo, North Macedonia, Montenegro and Serbia), highlighting that although these countries have strengthened their competitiveness through economic reforms over the past decades, significant challenges remain, with structural weaknesses further amplified by the COVID-19 pandemic, underscoring the need for continued policy implementation and upgrading.

In summary, this study highlights substantial geographical inequalities in healthy ageing across Europe, with SEE and CEE countries consistently experiencing less favourable outcomes compared to Western and Northern regions. While improvements in healthy life expectancy were observed in many countries prior to 2019, these gains were disrupted in recent years, likely reflecting the impact of the COVID-19 pandemic. Importantly, the observed inequalities among adults aged 70 years and older reflect cumulative life-course exposures, including differences in education, income, health behaviours, and access to preventive care, rather than health system performance in old age alone [49]. Together, these findings underscore the need for integrated, life-course and region-specific public health strategies to effectively reduce disparities and support healthy ageing across Europe.

## Limitations

A major strength of this study is the use of population-based data to provide comprehensive assessment of healthy ageing capturing health and socioeconomic dimensions among older adults across 33 countries in Europe. Nonetheless, several limitations should be acknowledged. Firstly, as this is a descriptive comparative study, our findings cannot support causal inferences regarding the determinants of healthy ageing. Secondly, this ecological analysis was based on aggregated, country-level data, which may cover important within-country and regional variability related to data collection methods, lifestyle behaviours, and healthcare access. Moreover, this analysis did not differentiate between national healthcare system models, which can significantly impact access, organization, and outcomes of NCD care. Furthermore, the analysis was based on data from 2011 to 2021. While this timeframe provided sufficient temporal depth to observe trends, extending the study period could enhance the robustness and stability of the findings. Additional indicators for which complete data was not available across all countries and years were omitted from the analysis. Finally, as our analysis extends to 2021, key indicators may be influenced by short‑term effects of COVID‑19 pandemic such as excess mortality, that do not reflect underlying structural health trends [50–52]. Despite these limitations, the used data sources and analytical approach were adequate for identifying disparities and temporal patterns in healthy ageing indicators.

## Conclusion

SEE countries consistently demonstrate weaker socioeconomic performance and disproportionate burden of age-related diseases compared to most EU counterparts. However, in certain key indicators such as healthy life expectancy some SEE countries demonstrate similar results to those in Central and Eastern European countries. Our findings indicate the importance of combining regionally coordinated policies with country-specific strategies to effectively promote healthy ageing across both EU and non-EU nations and to help narrow persistent gaps in ageing trajectories and socioeconomic development between European countries.

## Supplementary Information

Below is the link to the electronic supplementary material.

Supplementary file1 (DOCX 1039 kb)

## Data Availability

Publicly available datasets were analysed in this study. Data can be found here: http://ghdx.healthdata.org/gbd-results-tool, https://www.who.int/data/gho/data/indicators and http://hdr.undp.org/en/data.
