# Supplementary material for: Unequal healthy ageing trajectories across Europe: socioeconomic development and age-related disease burden in the European Union and South-Eastern European countries
Source: GeroScience. 2026 Apr 29;48(3):3883–97. doi: 10.1007/s11357-026-02273-0 (PMC13356215; doi:10.1007/s11357-026-02273-0)
Supplement: Supplementary file 1 — Supplementary file1 (DOCX 1039 kb) [file 11357_2026_2273_MOESM1_ESM.docx]

**Unequal Healthy Ageing Trajectories Across Europe: Socioeconomic Development and Age-Related Disease Burden in the European Union and South-Eastern European Countries**

Nora Kovacs^1,2^, Peter Piko^1,3^, David Major^4,5,6^, Vince Fazekas-Pongor^4,5,6^, Zoltan Ungvari^7,8^, Roza Adany^1,2,3,4^

^1^ Department of Public Health and Epidemiology, Faculty of Medicine, University of Debrecen, Debrecen, Hungary

^2^ HUN-REN–UD Public Health Research Group, Department of Public Health and Epidemiology, Faculty of Medicine, University of Debrecen, Debrecen, Hungary

^3^ National Laboratory for Health Security, Center for Epidemiology and Surveillance, Semmelweis University, Budapest, Hungary

^4^ Institute of Preventive Medicine and Public Health, Semmelweis University, Budapest, Hungary

^5^ Jozsef Fodor Center for Prevention and Healthy Aging, Semmelweis University, Budapest, Hungary

^6^ Outpatient Clinic, Semmelweis University, Budapest, Hungary

^7^ International Training Program in Geroscience, Doctoral College, Health Sciences Program/Institute of Preventive Medicine and Public Health, Semmelweis University, Budapest, Hungary

^8^ Vascular Cognitive Impairment, Neurodegeneration and Healthy Brain Aging Program, Department of Neurosurgery, University of Oklahoma Health Sciences Center, Oklahoma City, OK, USA

corresponding author

Roza Adany, MD, PhD

Institute of Preventive Medicine and Public Health, Semmelweis University, Budapest, Hungary

[adany.roza@med.unideb.hu](mailto:adany.roza@med.unideb.hu) or [adany.roza@semmelweis.hu](mailto:adany.roza@semmelweis.hu)


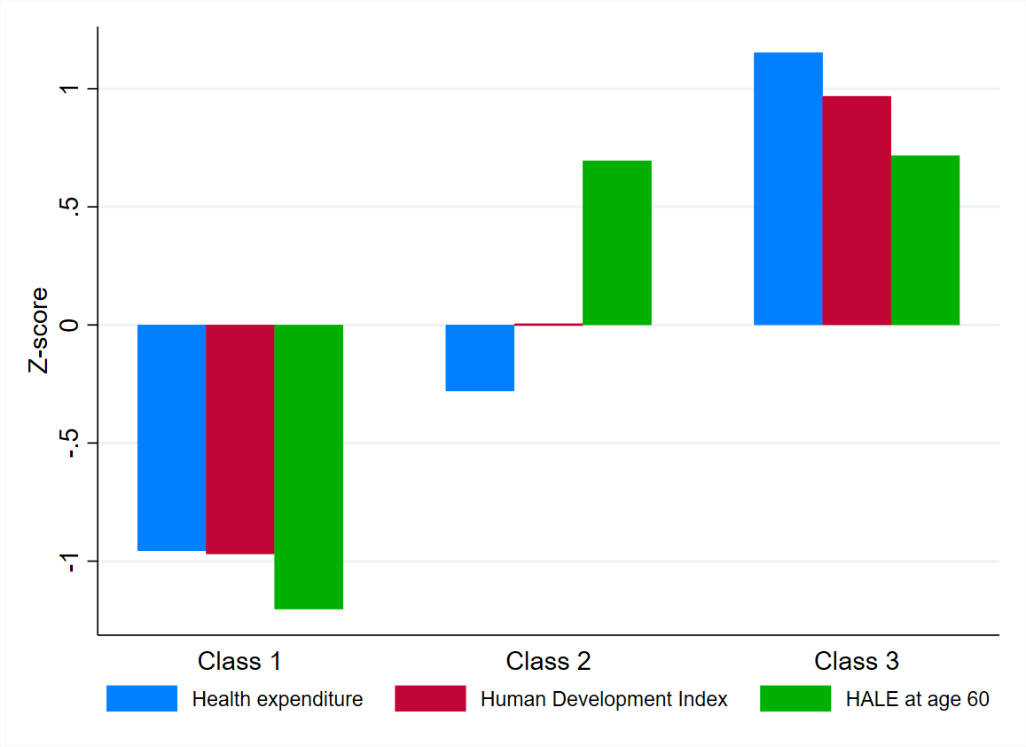


Figure S1 Latent profile analysis of EU member states based on health expenditure, socioeconomic development, and healthy life expectancy at age 60

Table S1 Means and pairwise comparisons of health and socioeconomic characteristics across EU clusters and SEE countries including Israel

|  | **Mean** | | | |  |
| --- | --- | --- | --- | --- | --- |
| **Indicator** | **Cluster 1** | **Cluster 2** | **Cluster 3** | **SEE countries** | **p-value#** |
| **Current health expenditure per capita, in international dollar (PPP)** | 7097.86* | 4253.02* | 3115.41 | 2087.44 | <0.001** |
| **Human Development Index** | 0.94* | 0.90 | 0.87 | 0.81 | <0.001** |
| Life expectancy at birth (years) | 81.87* | 81.46* | 74.78 | 74.69 | <0.001** |
| Expected years of schooling | 17.61* | 17.44* | 15.78* | 14.47 | 0.001** |
| Mean years of schooling | 12.66 | 11.39 | 12.72 | 11.56 | 0.046** |
| GNI per capita **in international dollar (PPP)** | 67313.76* | 42635.57* | 38654.61* | 23402.99 | <0.001** |
| **HALE at birth (years)** | 70.15* | 69.97 | 64.97 | 65.20 | <0.001** |
| **HALE at 60 (years)** | 18.19* | 18.02* | 14.40 | 14.23 | <0.001** |
| **Death rate per 100,000 people (aged 70+)** |  |  |  |  |  |
| All-cause mortality | 5154.03* | 5689.67 | 8077.55 | 8662.41 | <0.001** |
| Alzheimer's disease and other dementias | 443.74* | 462.98* | 324.99 | 283.45 | <0.001** |
| Colon and rectum cancer | 172.66 | 178.02 | 214.93* | 160.78 | 0.017** |
| Diabetes mellitus | 108.05 | 164.06 | 138.53 | 220.76 | 0.179 |
| Ischemic heart disease | 756.11* | 711.45 | 1945.87 | 1902.86 | <0.001** |
| Stroke | 402.45* | 612.69 | 1079.00 | 1782.69 | <0.001** |
| Tracheal, bronchus, and lung cancer | 230.52 | 216.87 | 216.26 | 219.57 | 0.863 |
| **YLD rate per 100,000 people (aged 70+)** |  |  |  |  |  |
| All-cause YLD | 27235.41 | 27534.63 | 28310.03 | 27393.89 | 0.136 |
| Alzheimer's disease and other dementias | 1969.92 | 2128.79* | 1839.99 | 1682.99 | 0.045** |
| Colon and rectum cancer | 175.96* | 156.40* | 134.06* | 90.49 | 0.001** |
| Diabetes mellitus | 1421.49* | 2035.25 | 2012.84 | 2480.97 | 0.003** |
| Ischemic heart disease | 436.34 | 372.86* | 605.28 | 559.65 | 0.001** |
| Stroke | 957.69 | 747.36 | 1191.76 | 1071.87 | 0.047** |
| Tracheal, bronchus, and lung cancer | 53.06 | 46.65 | 44.86 | 42.12 | 0.394 |
| **Population ages 65 and above (% of total population)** | 19.40 | 20.65 | 20.10 | 16.91 | 0.165 |
| **Urban population (% of total population)** | 83.72* | 70.26 | 65.47 | 61.62 | 0.007** |
| **Countries (n=34)** | Austria | Cyprus | Bulgaria | Albania |  |
|  | Belgium | Spain | Czechia | Bosnia and Herzegovina |  |
|  | Germany | Greece | Estonia | Israel |  |
|  | Denmark | Italy | Croatia | Moldova |  |
|  | Finland | Portugal | Hungary | North Macedonia |  |
|  | France | Slovenia | Lithuania | Montenegro |  |
|  | Ireland |  | Latvia | Serbia |  |
|  | Luxembourg |  | Poland |  |  |
|  | Malta |  | Romania |  |  |
|  | Netherlands |  | Slovakia |  |  |
|  | Sweden |  |  |  |  |
|  | n=11 | n=6 | n=10 | n=7 |  |

|  | **Health expenditure** | **Human Development Index** | **Healthy life expectancy at age 60** |
| --- | --- | --- | --- |
|  | *a* | *b* | *c* |
| **Alzheimer’s disease** | r=0.70, p<0.001 | r=0.75, p<0.001 | r=0.68, p<0.001 |
|  | 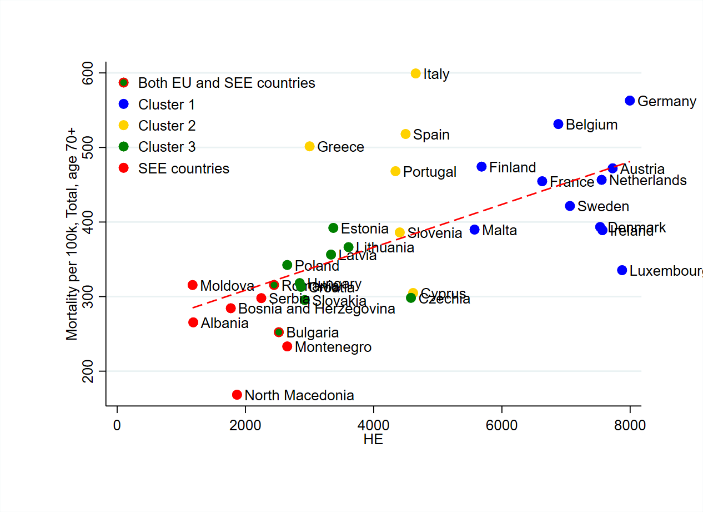 | 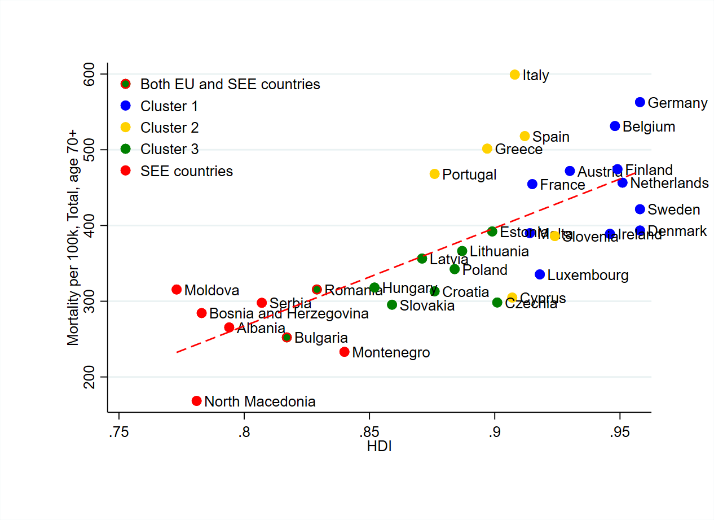 | 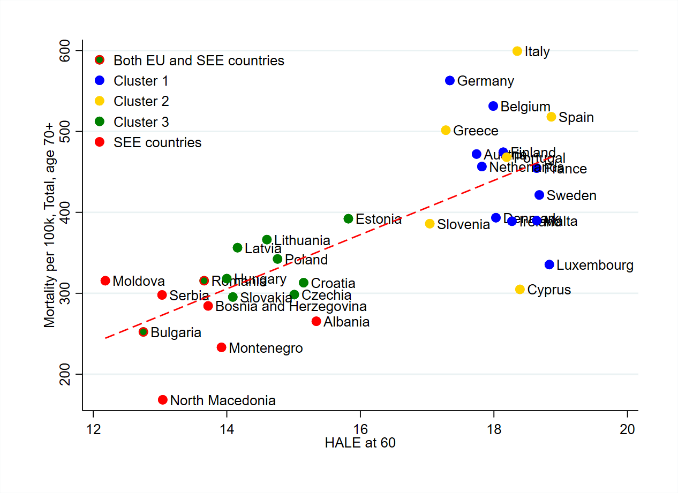 |
|  | *a* | *b* | *c* |
| **Colorectal cancer** | r=–0.20, p=0.262 | r=–0.15, p=0.410 | r=–0.25, p=0.162 |
|  | 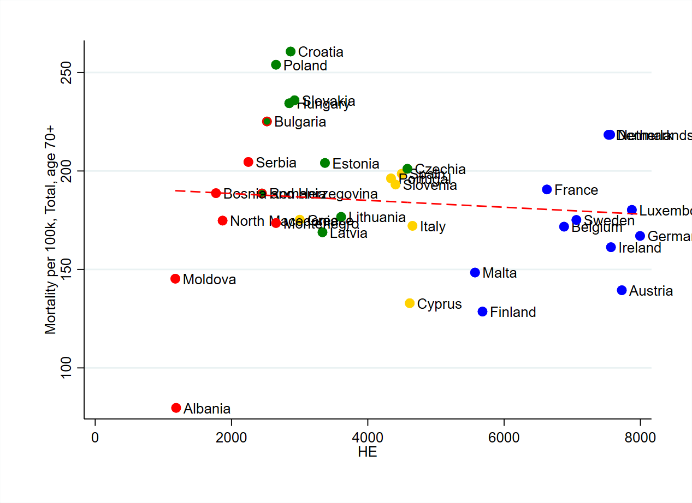 | 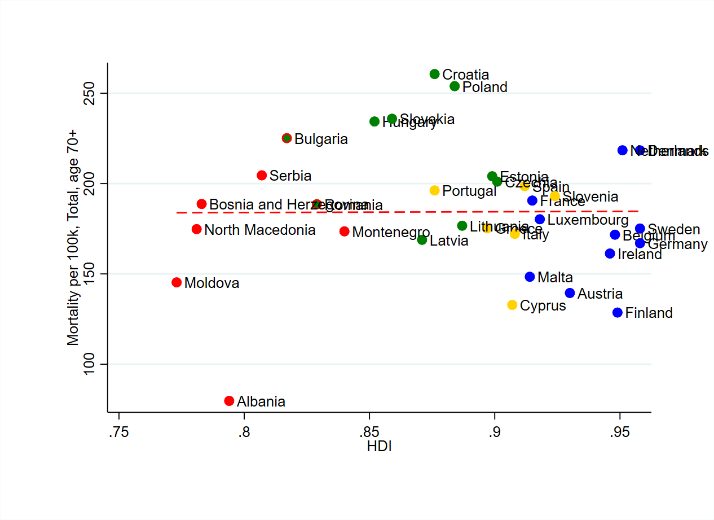 | 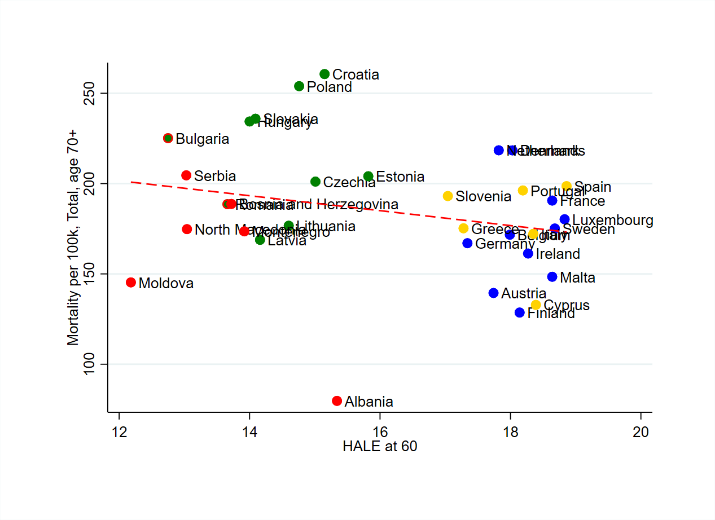 |
|  | *d* | *e* | *f* |
|  |  |  |  |
|  |  |  |  |
|  |  |  |  |
|  |  |  |  |
| **Diabetes mellitus** | r=–0.20, p=0.273 | r=–0.28, p=0.118 | r=–0.18, p=0.328 |
|  | 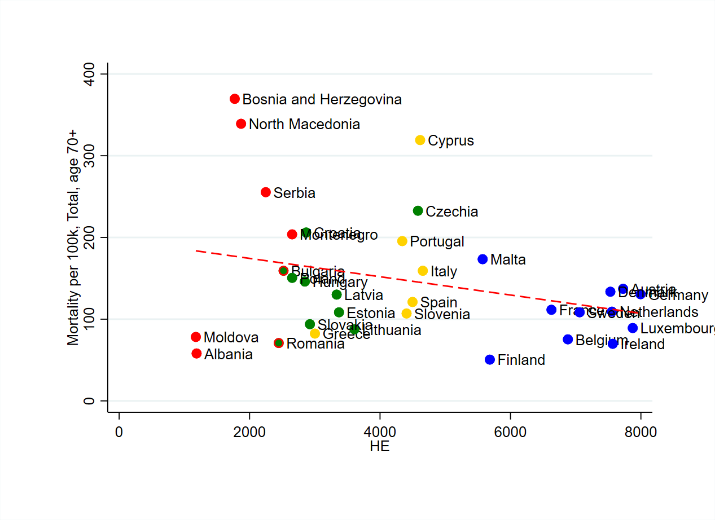 | 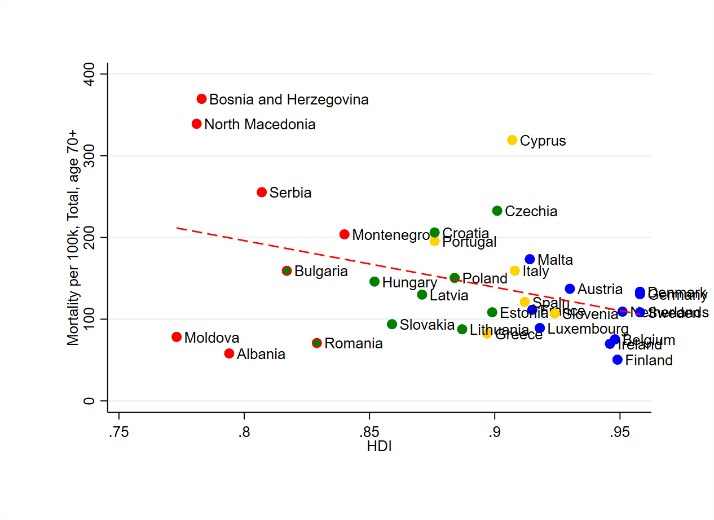 | 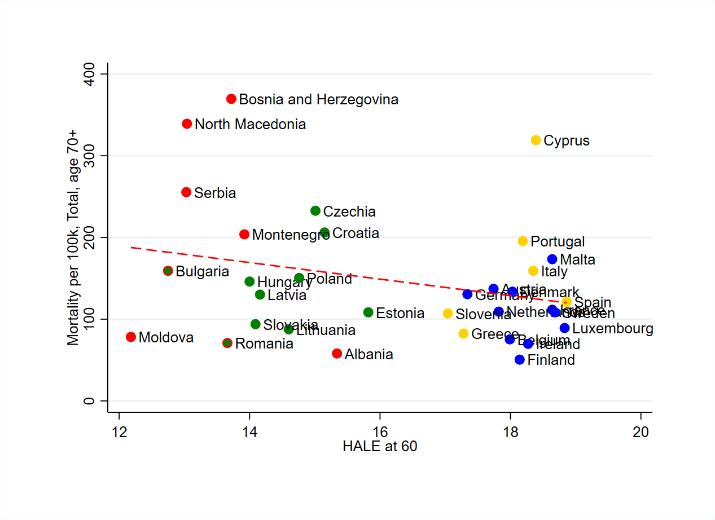 |
|  | *g* | *h* | *i* |
| **Ischemic heart disease** | r=–0.73, p<0.001 | r=–0.74, p<0.001 | r=–0.84, p<0.001 |
|  | 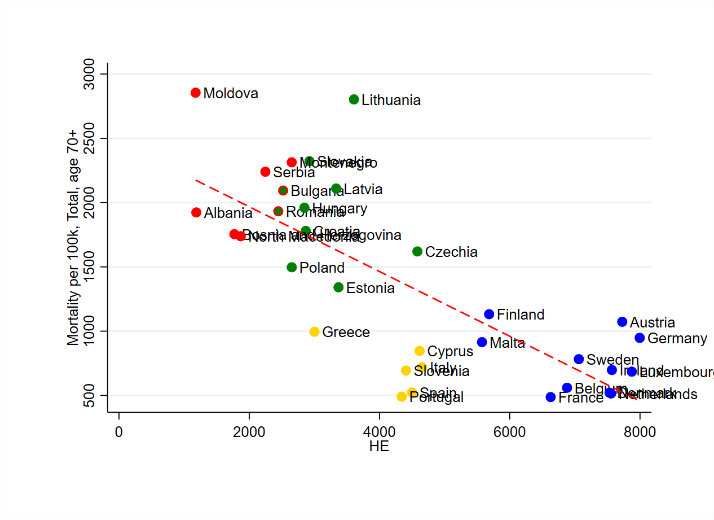 | 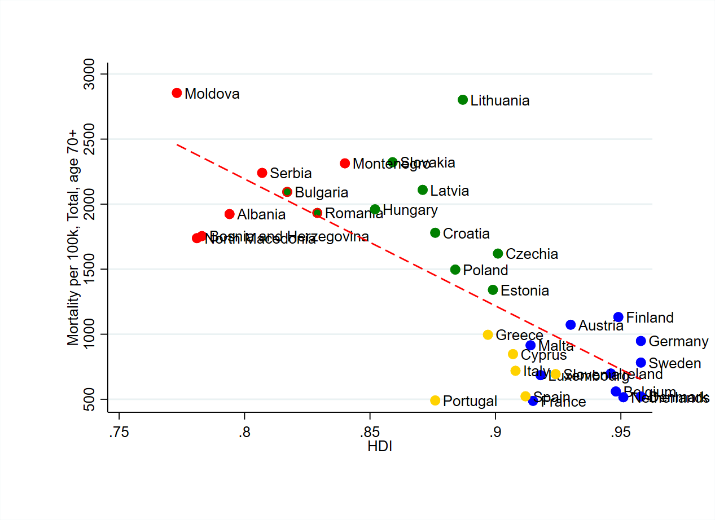 | 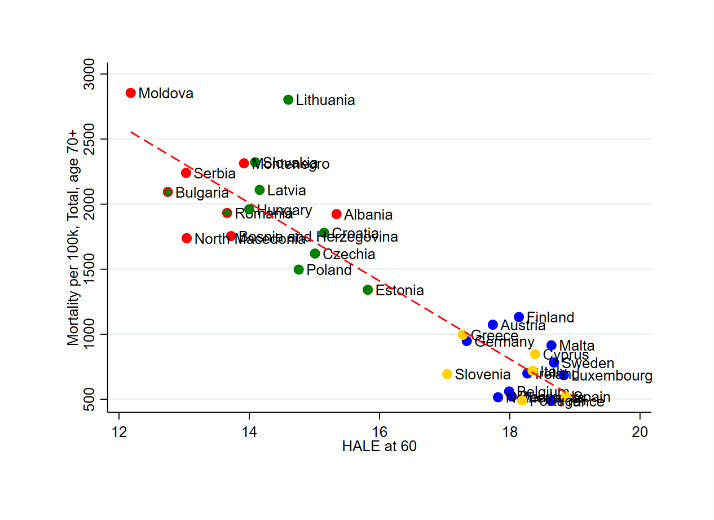 |
|  | *j* | *k* | *l* |
|  |  |  |  |
|  |  |  |  |
|  |  |  |  |
|  |  |  |  |
|  |  |  |  |
|  |  |  |  |
| **Stroke** | r=–0.88, p<0.001 | r=–0.87, p<0.001 | r=–0.82, p<0.001 |
|  | 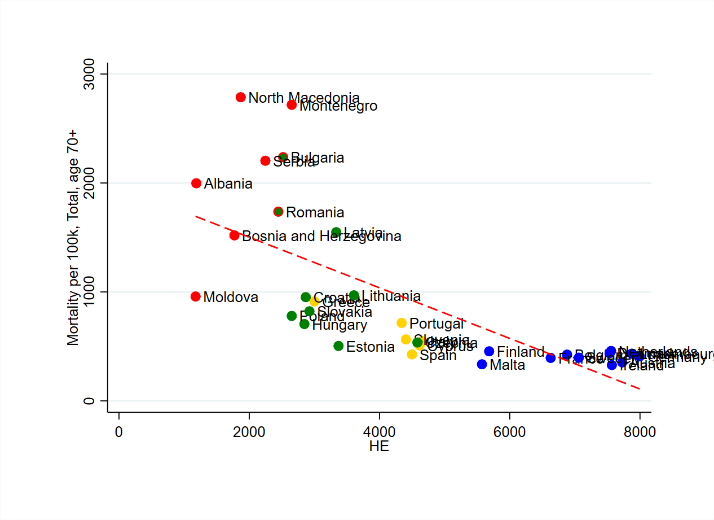 | 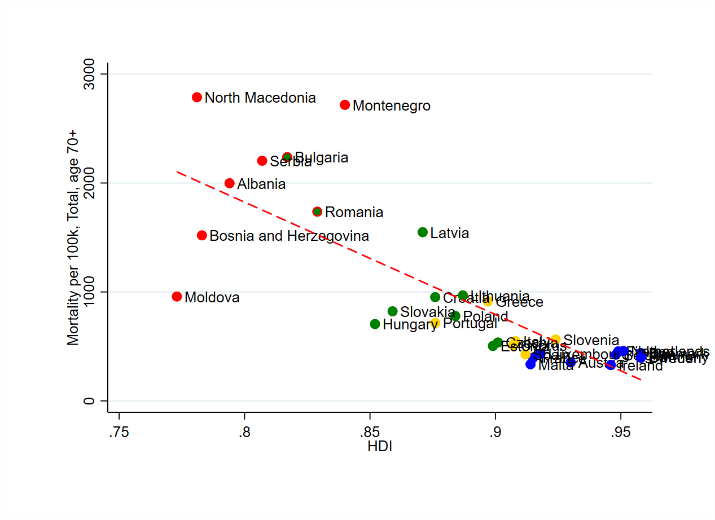 | 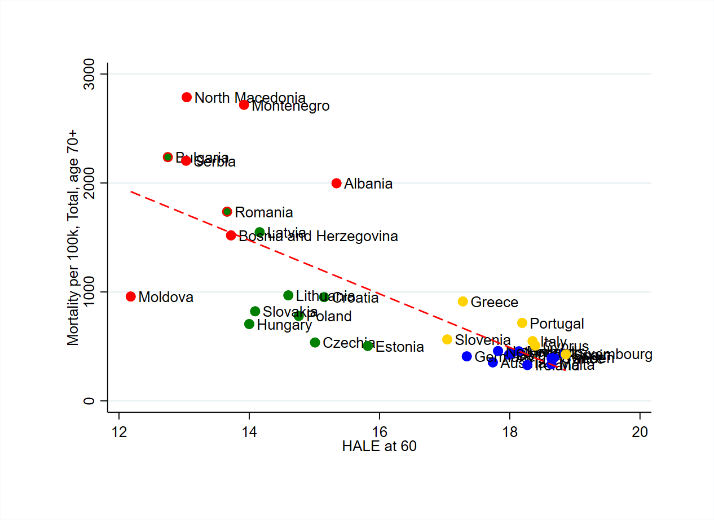 |
|  | *m* | *n* | *o* |
| **Lung cancer** | r=0.07, p=0.706 | r=0.16, p=0.360 | r=–0.03, p=0.867 |
|  | 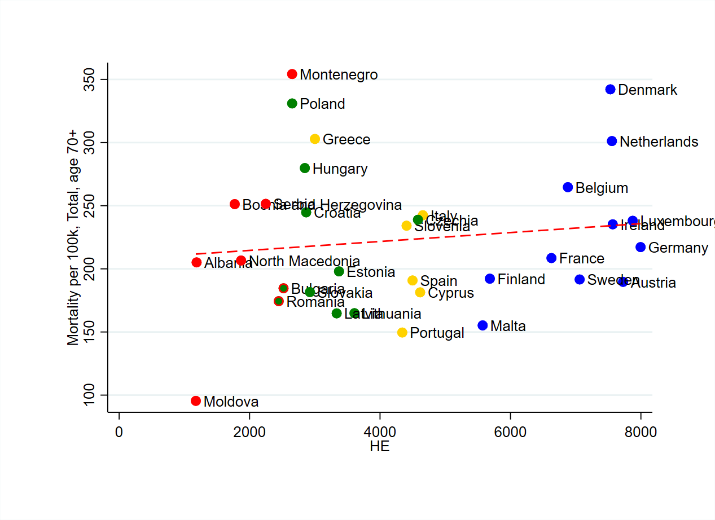 | 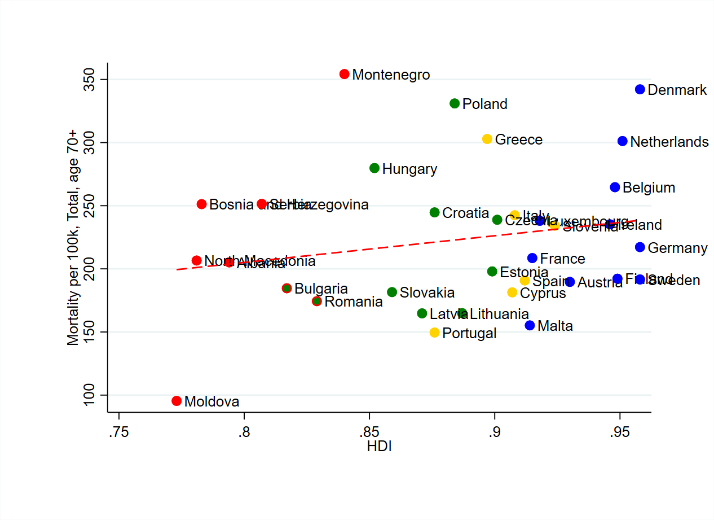 | 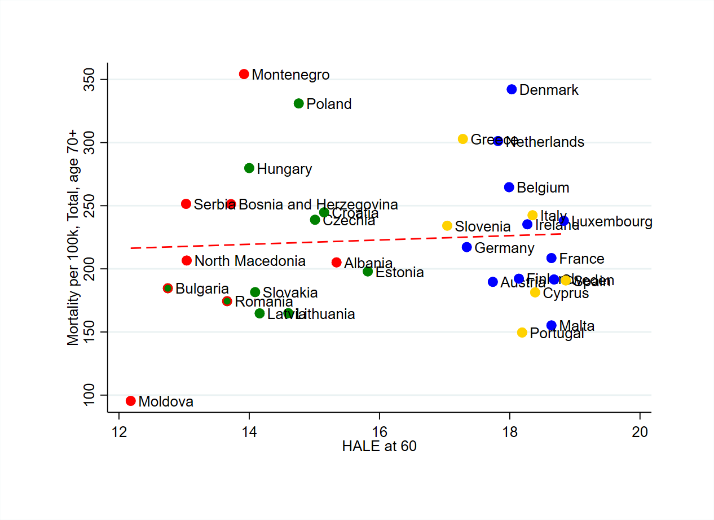 |
|  | *p* | *q* | *r* |

Figure S2. Spearman correlations between health and socioeconomic indicators and cause-specific mortality and YLD rates among aged 70+ for European countries (n=33) in 2021. HE: Health expenditure, HDI: Human Development Index, HALE: Healthy life expectancy.

Table S2 Trends in mortality rates among adults aged 70+ in European countries, 2011–2021, estimated by Joinpoint regression

| **Countries** | **Trend 1** | **APC** | **p-value** | **Trend 2** | **APC** | **p-value** | **AAPC, 2011-2021** | **p-value** |
| --- | --- | --- | --- | --- | --- | --- | --- | --- |
| Albania | 2011-2019 | 1.79 | 0.093 | 2019-2021 | 9.37* | <0.001 | 3.26* | <0.001 |
| Austria | 2011-2019 | 0.26 | 0.647 | 2019-2021 | 3.22* | 0.009 | 0.84* | 0.004 |
| Belgium | 2011-2013 | 0.93 | 0.655 | 2013-2021 | -0.12 | 0.722 | 0.09 | 0.888 |
| Bosnia and Herzegovina | 2011-2019 | 1.63 | 0.100 | 2019-2021 | 8.30* | <0.001 | 2.93* | <0.001 |
| Bulgaria | 2011-2019 | -1.11* | <0.001 | 2019-2021 | 16.75* | <0.001 | 2.23* | <0.001 |
| Croatia | 2011-2019 | 0.22 | 0.457 | 2019-2021 | 7.45* | <0.001 | 1.63 | <0.001 |
| Cyprus | 2011-2016 | -3.67* | <0.001 | 2016-2021 | -0.95 | 0.079 | -2.32* | <0.001 |
| Czechia | 2011-2019 | -1.63* | <0.001 | 2019-2021 | 9.21* | <0.001 | 0.45 | 0.091 |
| Denmark | 2011-2019 | -2.35* | <0.001 | 2019-2021 | 1.53 | 0.218 | -1.59* | <0.001 |
| Estonia | 2011-2019 | -0.23 | 0.422 | 2019-2021 | 7.42* | 0.006 | 1.26* | 0.027 |
| Finland | 2011-2013 | 0.48 | 0.930 | 2013-2021 | -1.63 | 0.014 | -1.21* | <0.001 |
| France | 2011-2019 | 0.13 | 0.827 | 2019-2021 | 1.45 | 0.221 | 0.39 | 0.160 |
| Germany | 2011-2016 | 1.55* | 0.002 | 2016-2021 | 0.84 | 0.254 | 1.20* | <0.001 |
| Greece | 2011-2019 | 1.09* | <0.001 | 2019-2021 | 7.15* | <0.001 | 2.27* | <0.001 |
| Hungary | 2011-2019 | -0.58* | <0.001 | 2019-2021 | 6.46* | <0.001 | 0.79* | 0.000 |
| Ireland | 2011-2016 | -1.04 | 0.112 | 2016-2021 | -2.34* | <0.001 | -1.69* | <0.001 |
| Italy | 2011-2019 | 0.42 | 0.858 | 2019-2021 | 4.00 | 0.086 | 1.13* | 0.027 |
| Latvia | 2011-2019 | 0.05 | 0.980 | 2019-2021 | 9.70* | <0.001 | 1.91* | <0.001 |
| Lithuania | 2011-2019 | 0.24 | 0.305 | 2019-2021 | 9.91* | <0.001 | 2.10* | <0.001 |
| Luxembourg | 2011-2015 | 0.95 | 0.124 | 2015-2021 | -0.80 | 0.062 | -0.11 | 0.601 |
| Malta | 2011-2019 | -3.53* | 0.026 | 2019-2021 | 3.52 | 0.376 | -2.16 | <0.001 |
| Moldova | 2011-2019 | -1.16* | 0.011 | 2019-2021 | 10.88* | 0.001 | 1.14* | 0.044 |
| Montenegro | 2011-2019 | 2.39* | 0.039 | 2019-2021 | 13.60* | <0.001 | 4.54* | <0.001 |
| Netherlands | 2011-2019 | -0.90* | 0.015 | 2019-2021 | 3.22 | 0.068 | -0.09 | 0.553 |
| North Macedonia | 2011-2019 | -0.45 | 0.063 | 2019-2021 | 9.92* | <0.001 | 1.54* | <0.001 |
| Poland | 2011-2019 | 0.02 | 0.986 | 2019-2021 | 9.75* | <0.001 | 1.89 | <0.001 |
| Portugal | 2011-2019 | -0.42* | 0.004 | 2019-2021 | 3.41* | 0.000 | 0.33* | 0.036 |
| Romania | 2011-2019 | 0.33 | 0.147 | 2019-2021 | 10.40* | <0.001 | 2.27* | <0.001 |
| Serbia | 2011-2019 | -0.65* | 0.022 | 2019-2021 | 11.43* | <0.001 | 1.65 | <0.001 |
| Slovakia | 2011-2019 | -1.74* | <0.001 | 2019-2021 | 11.31 | <0.001 | 0.74 | 0.129 |
| Slovenia | 2011-2014 | -1.70 | 0.412 | 2014-2021 | 2.00 | 0.060 | 0.87 | 0.068 |
| Spain | 2011-2018 | 0.33 | 0.864 | 2018-2021 | 1.53 | 0.354 | 0.69 | 0.122 |
| Sweden | 2011-2018 | -2.49* | 0.006 | 2018-2021 | 0.19 | 0.881 | -1.69* | <0.001 |

APC: annual percentage change, AAPC: average annual percentage change. *statistically significant (p<0.05)

Table S3 Trends in YLD rates among adults aged 70+ in European countries, 2011–2021, estimated by Joinpoint regression

| **Countries** | **Trend 1** | **APC** | **p-value** | **Trend 2** | **APC** | **p-value** | **AAPC, 2011-2021** | **p-value** |
| --- | --- | --- | --- | --- | --- | --- | --- | --- |
| Albania | 2011-2019 | 0.01 | 0.509 | 2019-2021 | 1.17* | <0.001 | 0.24* | <0.001 |
| Austria | 2011-2017 | 0.14** | 0.015 | 2017-2021 | 0.00 | 0.973 | 0.09* | <0.001 |
| Belgium | 2011-2014 | 0.25* | 0.028 | 2014-2021 | -0.15* | 0.003 | -0.03 | 0.276 |
| Bosnia and Herzegovina | 2011-2015 | 0.59* | <0.001 | 2015-2021 | 0.17 | 0.151 | 0.34* | <0.001 |
| Bulgaria | 2011-2019 | -0.08* | 0.052 | 2019-2021 | 1.03* | <0.001 | 0.14* | 0.004 |
| Croatia | 2011-2015 | 0.51* | <0.001 | 2015-2021 | 0.23 | 0.166 | 0.34* | <0.001 |
| Cyprus | 2011-2017 | -0.41* | <0.001 | 2017-2021 | 0.13 | 0.156 | -0.20* | <0.001 |
| Czechia | 2011-2019 | -0.34* | 0.018 | 2019-2021 | 0.42 | 0.301 | -0.19* | <0.001 |
| Denmark | 2011-2017 | -0.29* | <0.001 | 2017-2021 | 0.15* | <0.001 | -0.11* | <0.001 |
| Estonia | 2011-2017 | 0.48* | <0.001 | 2017-2021 | 0.22 | 0.083 | 0.38* | <0.001 |
| Finland | 2011-2019 | -0.54* | 0.010 | 2019-2021 | 0.00 | 0.722 | -0.43* | <0.001 |
| France | 2011-2014 | 0.33* | <0.001 | 2014-2021 | -0.05 | 0.052 | 0.06* | 0.018 |
| Germany | 2011-2018 | 0.84* | <0.001 | 2018-2021 | 0.10 | 0.316 | 0.61* | <0.001 |
| Greece | 2011-2019 | 0.24* | 0.017 | 2019-2021 | 0.35* | 0.008 | 0.26* | <0.001 |
| Hungary | 2011-2019 | -0.13* | <0.001 | 2019-2021 | 0.56* | <0.001 | 0.01 | 0.347 |
| Ireland | 2011-2019 | -0.23* | 0.001 | 2019-2021 | 0.09 | 0.455 | -0.16* | <0.001 |
| Italy | 2011-2014 | -0.13 | 0.250 | 2014-2021 | 0.46* | <0.001 | 0.29* | <0.001 |
| Latvia | 2011-2018 | 0.51* | <0.001 | 2018-2021 | 0.42* | <0.001 | 0.48* | <0.001 |
| Lithuania | 2011-2015 | -0.02 | 0.619 | 2015-2021 | -0.25* | <0.001 | -0.16* | <0.001 |
| Luxembourg | 2011-2018 | 0.00 | 0.985 | 2018-2021 | -0.19* | <0.001 | -0.06* | 0.008 |
| Malta | 2011-2018 | -0.14* | 0.034 | 2018-2021 | 0.44* | 0.022 | 0.04 | 0.454 |
| Moldova | 2011-2013 | 0.01 | 0.629 | 2013-2021 | 0.38 | 0.063 | 0.30* | <0.001 |
| Montenegro | 2011-2019 | 0.09 | 0.652 | 2019-2021 | 0.77* | 0.023 | 0.22* | <0.001 |
| Netherlands | 2011-2014 | 0.96* | <0.001 | 2014-2021 | -0.10* | 0.028 | 0.22* | <0.001 |
| North Macedonia | 2011-2019 | 0.02 | 0.851 | 2019-2021 | 0.85* | <0.001 | 0.18* | <0.001 |
| Poland | 2011-2019 | -0.07* | 0.005 | 2019-2021 | 0.76* | <0.001 | 0.09* | 0.002 |
| Portugal | 2011-2019 | -0.09* | <0.001 | 2019-2021 | 0.54* | <0.001 | 0.03* | 0.020 |
| Romania | 2011-2015 | 0.53* | <0.001 | 2015-2021 | 0.06* | 0.041 | 0.25* | <0.001 |
| Serbia | 2011-2019 | 0.10* | <0.001 | 2019-2021 | 1.25* | <0.001 | 0.33* | <0.001 |
| Slovakia | 2011-2019 | -0.33* | <0.001 | 2019-2021 | -0.08 | 0.234 | -0.28* | <0.001 |
| Slovenia | 2011-2015 | 0.11 | 0.128 | 2015-2021 | -0.06 | 0.145 | 0.00 | 0.896 |
| Spain | 2011-2014 | 0.53* | <0.001 | 2014-2021 | -0.09 | 0.050 | 0.10* | 0.014 |
| Sweden | 2011-2019 | -0.21* | <0.001 | 2019-2021 | 0.83* | 0.001 | 0.00 | 0.850 |

APC: annual percentage change, AAPC: average annual percentage change. *statistically significant (p<0.05)

Table S4 Trends in HALE at 60 in European countries, 2011–2021, estimated by Joinpoint regression

| **Countries** | **Trend 1** | **APC** | **p-value** | **Trend 2** | **APC** | **p-value** | **AAPC, 2011-2021** | **p-value** |
| --- | --- | --- | --- | --- | --- | --- | --- | --- |
| Albania | 2011-2018 | -0.17 | 0.618 | 2018-2021 | -2.36* | <0.001 | -0.83* | 0.000 |
| Austria | 2011-2019 | 0.23* | 0.048 | 2019-2021 | -1.18 | 0.054 | -0.05 | 0.693 |
| Belgium | 2011-2018 | 0.47 | 0.121 | 2018-2021 | -0.47 | 0.429 | 0.19 | 0.574 |
| Bosnia and Herzegovina | 2011-2019 | -0.15 | 0.098 | 2019-2021 | -5.83* | <0.001 | -1.31* | <0.001 |
| Bulgaria | 2011-2019 | 0.28* | 0.020 | 2019-2021 | -7.62* | <0.001 | -1.35* | <0.001 |
| Croatia | 2011-2019 | 0.42* | <0.001 | 2019-2021 | -3.36* | <0.001 | -0.35* | 0.001 |
| Cyprus | 2011-2019 | 0.31* | 0.006 | 2019-2021 | -0.33 | 0.289 | 0.18* | <0.001 |
| Czechia | 2011-2019 | 0.52* | <0.001 | 2019-2021 | -4.08* | <0.001 | -0.41* | <0.001 |
| Denmark | 2011-2014 | 0.82* | <0.001 | 2014-2021 | 0.23 | 0.144 | 0.41* | <0.001 |
| Estonia | 2011-2019 | 0.66* | <0.001 | 2019-2021 | -2.84* | 0.000 | -0.05 | 0.629 |
| Finland | 2011-2019 | 0.49* | 0.001 | 2019-2021 | -0.23 | 0.544 | 0.35 | <0.001 |
| France | 2011-2019 | 0.28* | 0.000 | 2019-2021 | -1.74* | <0.001 | -0.13 | 0.171 |
| Germany | 2011-2019 | -0.04 | 0.984 | 2019-2021 | -0.50 | 0.169 | -0.13 | 0.419 |
| Greece | 2011-2019 | 0.20 | 0.065 | 2019-2021 | -2.32* | 0.002 | -0.31* | 0.038 |
| Hungary | 2011-2019 | 0.24* | 0.014 | 2019-2021 | -3.75* | <0.001 | -0.57* | <0.001 |
| Ireland | 2011-2019 | 0.55* | <0.001 | 2019-2021 | -0.78* | 0.010 | 0.28* | <0.001 |
| Italy | 2011-2018 | 0.38 | 0.056 | 2018-2021 | -1.25* | 0.036 | -0.11 | 0.544 |
| Latvia | 2011-2019 | 0.60* | 0.000 | 2019-2021 | -4.19* | <0.001 | -0.38* | 0.028 |
| Lithuania | 2011-2019 | 0.69* | <0.001 | 2019-2021 | -3.67* | <0.001 | -0.20* | 0.004 |
| Luxembourg | 2011-2016 | 0.67* | <0.001 | 2016-2021 | 0.08 | 0.223 | 0.38* | <0.001 |
| Malta | 2011-2018 | 0.70* | <0.001 | 2018-2021 | -0.70* | <0.001 | 0.28* | <0.001 |
| Moldova | 2011-2019 | 1.39* | <0.001 | 2019-2021 | -8.22* | <0.001 | -0.61* | <0.001 |
| Montenegro | 2011-2018 | 0.62* | <0.001 | 2018-2021 | -3.98* | <0.001 | -0.78* | <0.001 |
| Netherlands | 2011-2019 | 0.32* | 0.046 | 2019-2021 | -1.62* | 0.050 | -0.07 | 0.705 |
| North Macedonia | 2011-2018 | 0.79* | <0.001 | 2018-2021 | -4.29* | <0.001 | -0.76* | <0.001 |
| Poland | 2011-2019 | 0.36* | 0.002 | 2019-2021 | -5.25* | <0.001 | -0.79* | <0.001 |
| Portugal | 2011-2018 | 0.40* | 0.042 | 2018-2021 | -0.41 | 0.280 | 0.16 | 0.111 |
| Romania | 2011-2019 | 0.25* | 0.004 | 2019-2021 | -5.84* | <0.001 | -1.00* | <0.001 |
| Serbia | 2011-2019 | 0.50* | <0.001 | 2019-2021 | -6.71* | <0.001 | -0.98* | <0.001 |
| Slovakia | 2011-2019 | 0.76* | <0.001 | 2019-2021 | -5.62* | <0.001 | -0.55* | 0.001 |
| Slovenia | 2011-2019 | 0.45* | <0.001 | 2019-2021 | -1.87* | 0.002 | -0.02 | 0.979 |
| Spain | 2011-2019 | 0.23 | 0.313 | 2019-2021 | -0.96 | 0.384 | -0.01 | 0.968 |
| Sweden | 2011-2019 | 0.28 | 0.288 | 2019-2021 | -0.11 | 0.827 | 0.20* | 0.030 |

APC: annual percentage change, AAPC: average annual percentage change. *statistically significant (p<0.05)

Table S5 Model fit statistics for latent class trajectory model

| **Class** | **Log-likelihood** | **AIC** | **BIC** |
| --- | --- | --- | --- |
| 1 | -52.53 | 117.06 | 126.04 |
| **2** | **-41.07** | **100.13** | **113.60** |
| 3 | -39.42 | 102.84 | 120.80 |

BIC: Bayesian Information Criterion, AIC: Akaike Information Criterion

Table S6 Classification and mean posterior probabilities of countries by trajectory class

| **Class** | **n** | **Countries** | **Mean posterior probability** | |
| --- | --- | --- | --- | --- |
|  |  |  | **1** | **2** |
| Class 1 | 15 | Albania, Bosnia and Herzegovina, Bulgaria, Croatia, Czechia, Hungary, Latvia, Lithuania, Moldova, Montenegro, North Macedonia, Poland, Romania, Serbia, Slovakia | 0.999 | 0.001 |
| Class 2 | 18 | Austria, Belgium, Cyprus, Denmark, Estonia, Finland, France, Germany, Greece, Ireland, Italy, Luxembourg, Malta, Netherlands, Portugal, Slovenia, Spain, Sweden | 0.012 | 0.988 |


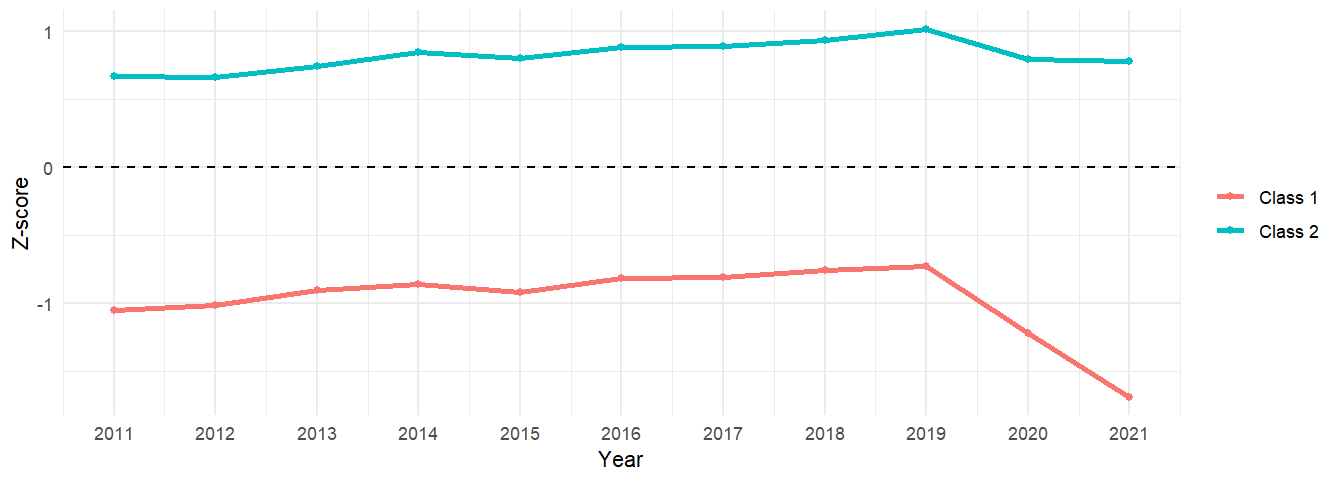


Figure S3 Latent class trajectories of healthy life expectancy at age 60
